# Supplementary material for: A Global Screen for Assembly State Changes of the Mitotic Proteome by SEC-SWATH-MS
Source: Cell Syst. 2020 Feb 26;10(2):133–155.e6. doi: 10.1016/j.cels.2020.01.001 (PMC7042714; doi:10.1016/j.cels.2020.01.001)

P62328 | TYB4_HUMAN | TMSB4X TB4X THYB4 TMSB4
Monomer MW [kDa]: 5.053 Monomer expected elution fraction: 66

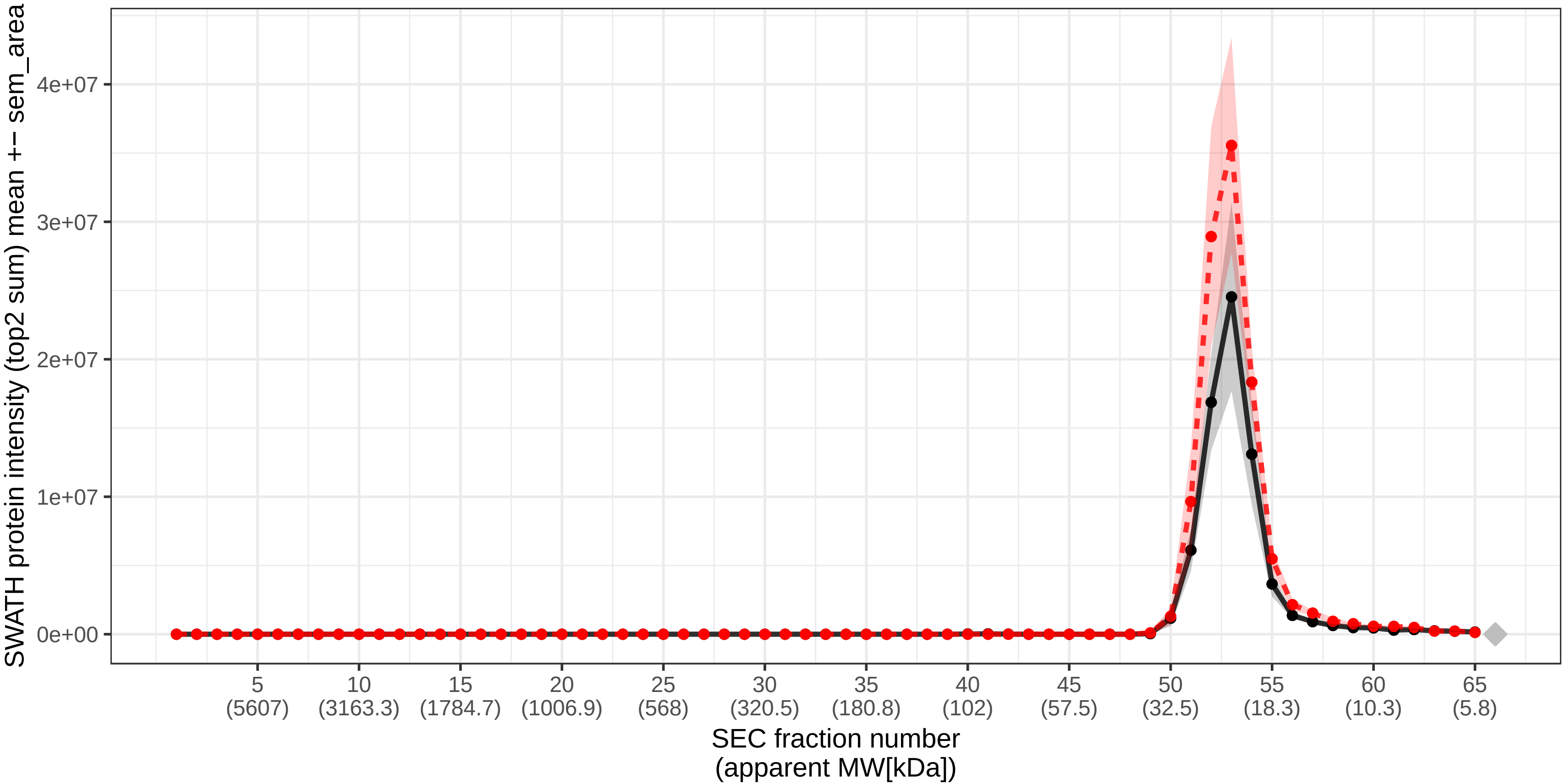

Supplement: Data S1. SEC-SWATH-MS Protein Chromatograms, Related to Figure 1 [file mmc6.zip › SECchrom_P62328_TYB4_HUMAN_TMSB4X_TB4X_THYB4_TMSB4.pdf]
